# Use of telemedicine by practising allergists before and during the SARS-CoV-2 pandemic: A survey among members of the Association of German Allergists (AeDA)

**DOI:** 10.1007/s40629-021-00175-5

**Published:** 2021-06-11

**Authors:** Stephanie Dramburg, Paolo Maria Matricardi, Ingrid Casper, Ludger Klimek

**Affiliations:** 1 Department of Pediatric Respiratory Medicine, Immunology and Critical Care Medicine, Charité—Universitätsmedizin Berlin, Augustenburger Platz 1, 13353 Berlin, Germany; 2 Centre for Rhinology and Allergology Wiesbaden, Wiesbaden, Germany

**Keywords:** Telemedicine, Video consultation, COVID-19, Allergology, Digitalisation

## Abstract

**Background:**

The severe acute respiratory syndrome coronavirus type 2 (SARS-CoV-2) pandemic presented unprecedented challenges to both inpatient and outpatient care. In order to maintain good care under necessary contact restrictions, especially in the outpatient sector, the use of telemedical applications was demanded and promoted. The exploratory survey among members of the Association of German Allergists (AeDA) was intended to show how these were received among allergists in private practice.

**Methods:**

The survey was restricted to actively practising members of the AeDA who had previously given their consent to receive such surveys (*n* = 437). They were invited by email to participate in a survey on the topic of “Telemedicine in everyday clinical practice in allergology”. The survey included quantitative and qualitative questions on the use of telemedicine services before and during the pandemic and was conducted anonymously on the SoSci Survey platform. Participation was possible in the period from June to August 2020.

**Results:**

In all, 76 specialists with additional qualification in allergology took part in the survey. Of these, 71 completed the full questionnaire. Before the start of the pandemic-related contact restrictions, 46.5% (33/71) stated that they had used telemedicine in their clinical practice. This number increased to 73.2% (52/71) after 31 January 2020. The largest increase (4.3% vs. 15.6%) was seen in the area of video consultations. Furthermore, 43/76 participants can imagine integrating telemedicine services into their daily clinical routine in the future.

**Conclusion:**

The use of telemedical services, especially video consultations, increased significantly during the SARS-CoV‑2 pandemic in Germany. The majority of respondents perceive the implementation as positive and can imagine continuing to use telemedical methods after the end of the pandemic.

## Introduction

The measures adopted by the federal government in spring 2020 to restrict the spread of severe acute respiratory syndrome coronavirus 2 (SARS-CoV-2) were widely implemented by the population, which initially led to a successful reduction in coronavirus disease 2019 (COVID-19) incidence. At the same time, however, a significant reduction in outpatient doctor–patient contacts became apparent, which posed new challenges for regular medical care in many respects. Regionally, a decrease in contacts of up to 75% could be observed with the same physician presence [1]. In order to continue to ensure continuous care for patients under contact restrictions and in times of concern about the risk of infection, the German Federal Ministry of Health called for and promoted the use of telemedical applications, especially video consultation. After the ban on exclusive remote treatment had already been relaxed in 2018 [2], the National Association of Statutory Health Insurance (SHI) Physicians now published simplified instructions for the implementation of telemedical services [3] including lists of certified technology providers [4] and corresponding remuneration structures [5]. While a survey by Stiftung Gesundheit described a clear increase in the use of telemedicine applications in 2020 [6], a survey of different participants in the health care system showed that the necessity, processes and benefits in different care structures differ significantly in some cases [7]. Although the authors also show a high acceptance of telemedical services across all professions, their implementation in different care scenarios seems to be confronted with different hurdles. For example, respondents from private clinics were more positive about the potential of telemedicine than their colleagues in private practices. In order to describe the implementation and use of different telemedicine methods in the everyday clinical practice of allergology specialists, we conducted an online survey among members of the Association of German Allergologists (AeDA).

## Methods

Among AeDA members, those actively practising in the branch who had previously given consent to receive such surveys (*n* = 437) were invited by email and advertisement to voluntarily participate in the online survey on telemedicine service use behaviour between 17 June and 31 August 2020. The survey included questions on the age group, gender and specialty of the participants as well as on the use of telemedicine before and after the introduction of the pandemic-related contact restrictions. There was also a survey on the use of different technologies and a questionnaire on the desire to continue offering telemedicine applications in the future. Possible complications of telemedical offers were also surveyed and participants were asked to indicate who took the decision regarding the justifiability of remote treatment. The date for differentiating the periods before and after the pandemic-related contact restrictions was set at 31 January 2020. The cross-sectional survey was conducted anonymously and in compliance with data protection regulations using the web-based survey platform SoSci Survey [8]. The descriptive data analysis was carried out using Microsoft Excel, version 16.44.

## Results

Seventy-six specialists (17.4%) with an additional qualification in allergology took part in the survey; 71 questionnaires were completed in full. The participants were 52.6% female and 42.1% belonged to the age group 51–60 years (Table 1).

**Table 1:** Description of the survey participants

	*n*	%
**Total number of participants**	76	*100*
**Gender**		
Female	40	*52.6*
Male	36	*47.4*
**Age group (in years)**		
31–40	2	*2.6*
41–50	18	*23.7*
51–60	32	*42.1*
61–70	24	*31.6*
**Specialisation**^**a**^		
Allergology	48	*63.2*
Otorhinolaringology	32	*42.1*
Dermatology	24	*31.6*
Pneumology	9	*11.8*
Internal medicine^b^	2	*2.6*
Pediatrics	5	*6.6*
General medicine	4	*5.3*
Occupational Medicine	1	*1.3*
Other	7	*9.2*

^a^Multiple answers possible

^b^except pneumology

In addition to allergology (*n* = 48; 63.2%), otorhinolarynoglogy (*n* = 32; 42.1%) and dermatology (*n* = 24; 31.6%) were among the most frequent specialities. With regard to the use of telemedicine applications, 46.5% (33/71) stated that they had already made use of them before 31 January 2020. This figure increased significantly to 73.2% (52/71) for use after 31 January 2020. The specification of technologies used showed stable trends in telephone consultations before and after the pandemic-related restrictions (20.5%; 24/117 vs. 20.3%; 39/192 respectively), while the largest increase was observed in the area of video consultations (4.3%; 5/117 vs. 15.6%; 30/192). The use of other technologies, such as patient-centred apps or digital exchange with colleagues, was stable at a low level, with no significant differences before and after the onset of the SARS-CoV‑2 pandemic (Table 2).

**Table 2:** Use of telemedical applications and specification of the technologies used before and after 31 January 2020

	*Before 31 January 2020*		*After 31 January 2020*		
	*n*	%	*n*	%	*p*-value
**Use of telemedicine applications**^**a**^	33	46.5	52	73.2	**<** **0.005**
**Total**^**b**^	**117**	*100*	**192**	*100*	–
Video consultation	5	*4.3*	30	*15.6*	**<** **0.005**
Telephone consultations (> 10 min)	24	*20.5*	39	*20.3*	**0.01**
Data transfer via digital platforms	6	*5.1*	9	*4.7*	0.42
Data transfer via e‑mail	23	*19.7*	30	*15.6*	0.22
Digital doctor–patient communication	5	*4.3*	9	*4.7*	0.26
Apps for healthcare professionals	7	*6.0*	8	*4.2*	0.78
Therapy apps	4	*3.4*	4	*2.1*	1.00
Symptom monitoring apps	6	*5.1*	11	*5.7*	0.20
Exposure apps	13	*11.1*	15	*7.8*	0.67
Digital exchange with colleagues	11	*9.4*	18	*9.4*	0.15
Online appointment	9	*7.7*	16	*8.3*	0.12
Other	4	*3.4*	3	*1.6*	0.70

^*a*^*n total* *=* *71 complete records*

^b^Multiple answers possible

**Fig. 1:**
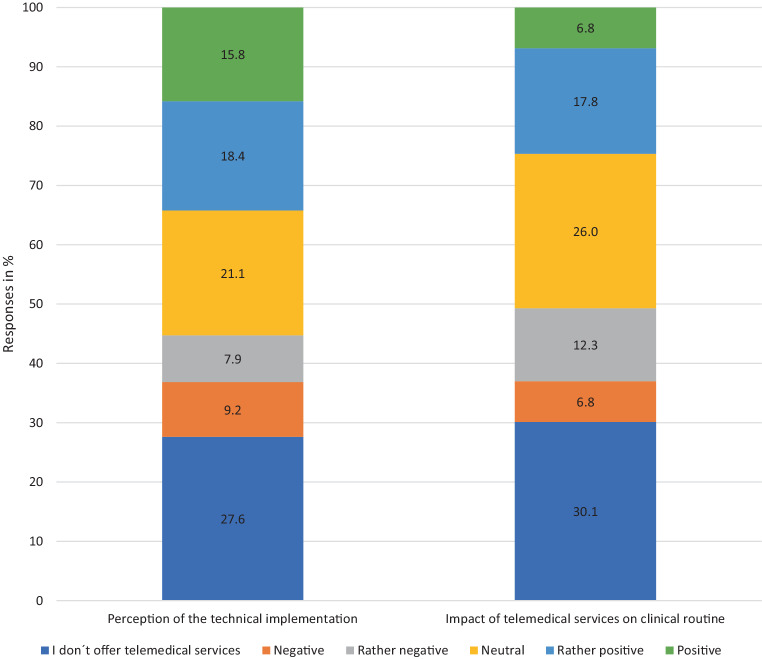
Perceived influence of telemedicine applications on everyday clinical life (The questions were completed by 76 and 73 participants respectively.)

The question about the perception of the technical implementation of telemedicine measures in their practice/clinic was answered by 55.3% of the respondents as “neutral”, “rather positive” or “positive” (21.1%; 18.4%; 15.8% respectively), while 50.6% of the participants gave this assessment with regard to the influence of telemedicine offers on their daily clinical routine (26% neutral; 17.8% rather positive; 6.8% positive) (Fig. 1).

**Fig. 2:**
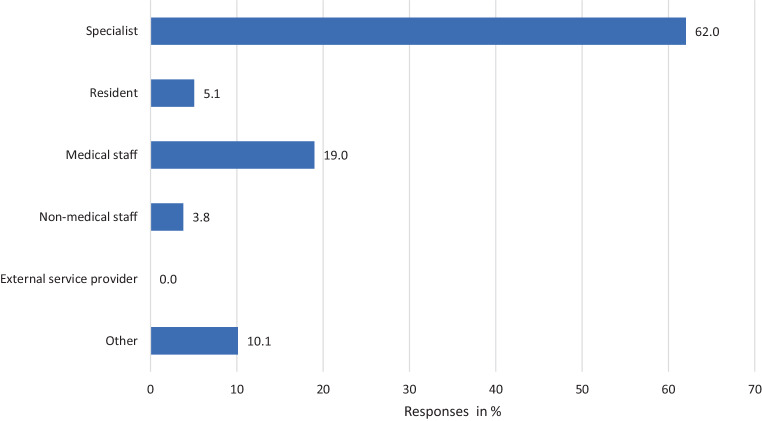
Decision-makers on the justifiability of telemedical counselling in individual cases

When asked who makes the decision on the justifiability of a (purely) telemedical consultation in individual cases, all participants answered that this decision is made within the clinic/practice itself. An external service provider was not entrusted with this decision among the respondents. In the majority of cases (62%), the specialists themselves decide whether the use of telemedical applications is appropriate or not (Fig. 2). Complications in connection with telemedical consultation were described by 9 of the 52 (17.3%) colleagues. Free-text statements on their quality revealed difficulties or delays in diagnostic decision-making as well as limitations in the clinical assessment of findings and communication problems with individual patients.

**Fig. 3:**
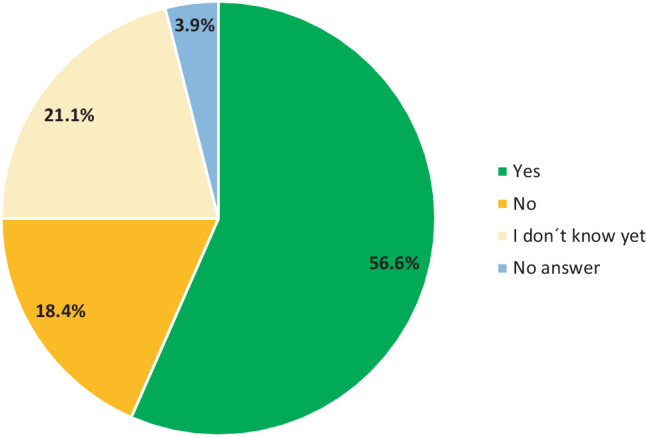
Statements on the desire to continue using telemedicine applications in the future (*n* = 76, figures in %)

When looking to the future, 56.6% of the respondents stated that they could imagine using telemedicine services in their daily clinical routine even after the current pandemic situation, while 21.1% were not yet able to make this decision conclusively and 18.4% rejected its use (Fig. 3). When asked for free comments and wishes regarding tele-allergology, respondents expressed a heterogeneous spectrum from enthusiastic support to strict rejection of telemedicine methods. However, participants repeatedly described a lack of time and personnel capacities for an additional telemedical offer or for dealing with its implementation.

## Discussion

The survey of practising allergists on the use of telemedicine in their daily clinical routine in times of the COVID‑19 pandemic showed a significant increase in the use of telemedical methods, especially with regard to video consultations.

The low response rate of 17.4% is most likely due to a certain fatigue regarding professional surveys around the COVID-19 pandemic and its impact on clinical practice. Although the majority of respondents were positive about the integration of telemedicine into everyday clinical practice, overall, a rather heterogeneous picture emerged in the free-text responses, which reflects the cross-section and corresponds to the data of Peine et al. collected in different care settings across professions [7].

It is also noteworthy that the decision on the justifiability of remote treatment is made in most cases by the specialists themselves and is not delegated to external service providers by any of the respondents. This should be evaluated in the light of the physician’s duty of care and liability [9], although the offer or scheduling of video consultation hours for defined scenarios by trained practice staff is conceivable, especially for known patients.

While the technical implementation of telemedical services is perceived as “neutral” to “positive” by the majority of participants, there is greater heterogeneity with regard to the influence of the new methods on everyday clinical practice. In free-text comments, it was repeatedly noted that the time-management during busy daily practice routine can be perceived as a challenge or that a lack of time and personnel resources can be a hurdle. Although the associations of SHI-accredited physicians have made support offers available, additional offers from the professional associations and practice-oriented training courses can further facilitate the integration of new technologies into existing structures.

Overall, more than half of the participants were in favour of continuing to offer telemedical consultations in the future, which is to be welcomed, especially with regard to specialist care in structurally weak regions.
